# What Is the Impact of mRNA 5′ TL Heterogeneity on Translational Start Site Selection and the Mammalian Cellular Phenotype?

**DOI:** 10.3389/fgene.2016.00156

**Published:** 2016-08-31

**Authors:** Joseph A. Curran, Benjamin Weiss

**Affiliations:** ^1^Department of Microbiology and Molecular Medicine, Medical School, University of GenevaGeneva, Switzerland; ^2^Institute of Genetics and Genomics of Geneva, University of GenevaGeneva, Switzerland

**Keywords:** mRNA, translation initiation, 5′TL (UTR), alternative promoters, transcriptional start site (TSS)

## Abstract

A major determinant in the efficiency of ribosome loading onto mRNAs is the 5′ TL (transcript leader or 5′ UTR). In addition, elements within this region also impact on start site selection demonstrating that it can modulate the protein readout at both quantitative and qualitative levels. With the increasing wealth of data generated by the mining of the mammalian transcriptome, it has become evident that a genes 5′ TL is not homogeneous but actually exhibits significant heterogeneity. This arises due to the utilization of alternative promoters, and is further compounded by significant variability with regards to the precise transcriptional start sites of each (not to mention alternative splicing). Consequently, the transcript for a protein coding gene is not a unique mRNA, but in-fact a complexed quasi-species of variants whose composition may respond to the changing physiological environment of the cell. Here we examine the potential impact of these events with regards to the protein readout.

## Introduction

The cellular phenotype is determined mainly by the cells protein composition. Events such as cell growth, differentiation, the stress response, apoptosis and even circadian regulation involve a re-seeding of the polysome with specific mRNA populations (Huang et al., 2013). The dynamic nature of this process has been known for a considerable time (Getz et al., 1976). More recently, the characterisation of ribosome-associated mRNAs (polysome profiling) has been employed for comparative cell typing (Doyle et al., 2008). The translational program can be modified rapidly, proceeding and, frequently orchestrating the later transcriptional response. Nonetheless, it is limited by the complexity of the existing transcriptome since it is from this pool that the mRNA will be recruited to seed the polysome.

With the advent of high-throughput RNA sequencing (RNA-seq) it has become increasingly popular to define the genetic expression profile of a cell by its transcriptome (Sultan et al., 2008; Eswaran et al., 2012). An approach in which a genes expression is estimated as the number of sequence reads assumes a linear 1 gene-1 RNA transcript-1 protein information transfer (1 gene = 1 protein). However, this is clearly a simplification. If one accepts that the cellular phenotype is dictated mainly by the proteome, it would be more judicious to analyze directly the cells protein composition, but, this is still technically difficult. The non-linear transfer of information from the genome to the proteome arises due to multiple layers of complexity. With regards to the mRNA transcript, this includes alternative splicing, multiple transcriptional start sites (TSS) and termination sites (TTS) all of which can be regulated in a cell-specific manner, and all serve to couple nuclear events to the protein readout in the cytoplasm (Davuluri et al., 2008; Pal et al., 2011). It is self-evident that alternative splicing events within the open reading frame (ORF) will alter the protein readout. However, with regards to alternative TSSs, which alter the nature of the first exon and the sequence of the mRNA 5′ untranslated region [UTR, also referred to as the transcript leader (TL); for why see below] this is not so intuitive. Nonetheless, the 5′ TL carries multiple elements that regulate the translational readout both quantitatively (amount of protein expressed) and qualitatively (the sequence of the proteins expressed). These are in-turn highly responsive to cellular signaling pathways that control proliferation, survival, and development (Davuluri et al., 2008). The regulatory elements include RNA structure, protein binding sites, internal ribosome entry sites (IRESes) and upstream AUG codons (uAUG) which may direct the translation of upstream open reading frames (uORFs; Pickering and Willis, 2005; Hinnebusch, 2011; see below).

### Regulation of Translation

**FIGURE 1:**
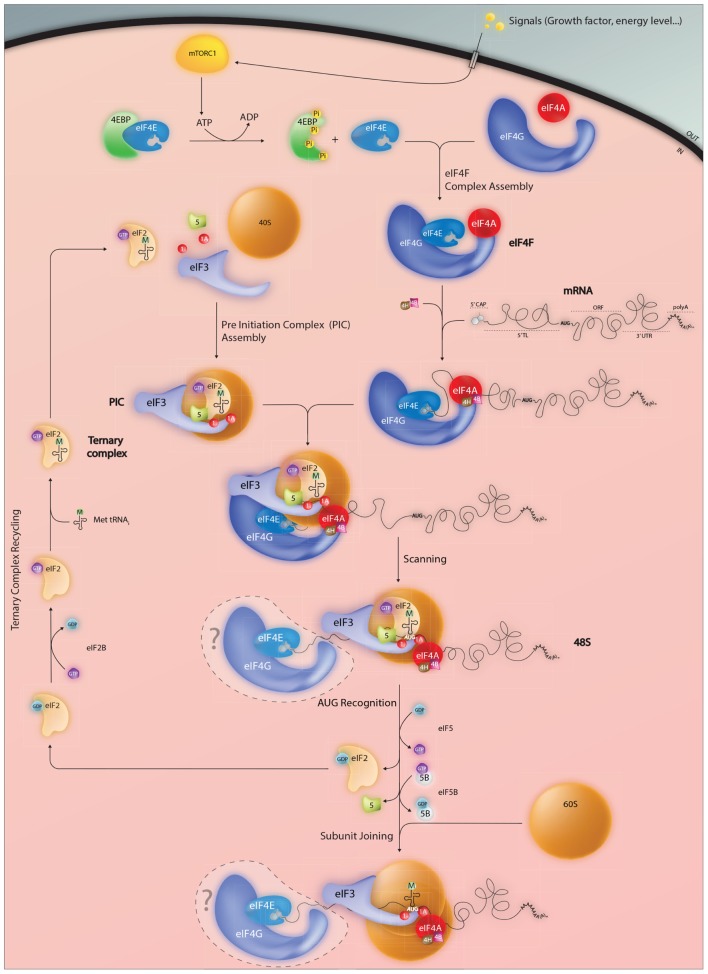
**Events impacting on the assembly of the initiation complex on the AUG start codon.** Mature mammalian mRNAs are exported from the nucleus into the cytoplasm carrying a distinctive 5′ cap structure. This is recognized by the eukaryotic initiation factor eIF4E which forms part of the trimolecular eIF4F complex. RNA structure close to the 5′ can limit cap accessibility and consequently plays an important role in modulating protein expression levels. In addition, active eIF4E levels are regulated via the 4E-binding proteins (4EBP) which in the hypophosphorylated state sequester eIF4E and prevent eIF4F assembly. Phosphorylation of 4EBP mediated by the mTORC1 kinase permits eIF4E release an event that serves to couple the protein readout to pro-proliferative signaling pathways. Once positioned at the 5′ cap, the eIF4F complex serves as a platform to recruit the pre-assembled 43S PIC thereby forming the 48S complex. Linear 5′–3′ ribosome scanning then occurs facilitated by the DEAD-box helicase eIF4A and its associated RNA binding proteins eIF4B and 4H. Exactly what happens to the eIF4E and 4G subunits during the scanning process (do they remain associated with the 5′ cap, move with the ribosome or are recycled) remain unclear. Start codon recognition (generally but not exclusively AUG) triggers GTP hydrolysis within both the eIF2.GTP.tRNAmet ternary complex (TC) and eIF5B.GTP that in-turn permits dis-assembly of the PIC and large 60S subunit joining. An active TC is regenerated via eIF2B a guanine nucleotide exchange factor.

Protein synthesis represents a key event in the regulation of gene expression. It can be subdivided into four main steps; initiation, elongation, termination, and sub-unit recycling. Most regulation is exerted at initiation, and this has been confirmed in translational profiling studies covering the entire mammalian transcriptome (Ingolia et al., 2011). Initiation in mammalian cells involves an interplay between a group of eukaryotic initiation factors (eIFs: see **Table 1** and **Figure 1**). The ternary complex (TC) composed of Met-tRNAi-eIF2-GTP is first loaded onto the 40S ribosomal subunit in combination with a series of eIFs. The Met-tRNAi is located in the P site in association with eIF2-GTP. This initiation factor is composed of tree subunits (α,β,γ). Its GTP loading is catalyzed by the Guanine Exchange Factor (GEF) eIF2B whose activity is also tightly regulated. The additional factors constituting the pre-initiation complex (PIC) include eIF1, eIF1A, eIF3, and eIF5. eIF1/1A are involved in the fidelity of start codon recognition with eIF1A positioned in the A-site and eIF1 near the P-site (Mitchell and Lorsch, 2008). The eIF5 interacts with both eIF2 and eIF3. It hydrolyses the eIF2 bound GTP to GDP, an event coupled to start site recognition. However, studies suggest that that this may also occur during scanning, with the hydrolysed inorganic phosphate (Pi) being retained within the PIC until start codon recognition (Algire et al., 2005). The eIF3 is the largest complex involved in PIC formation. It is composed of 13 different polypeptides in mammals (Damoc et al., 2007), participates in TC recruitment to the 40S and interacts with eIF1/1A and eIF5. It also prevents premature association of the 40S and 60S and is involved in PIC recruitment to the 5′ cap. The 60S associates with eIF6, which serves to block 40S association in the absence of mRNA (Ceci et al., 2003).

**Table 1:** List of eukaryotic initiation factors (eIFs) and their function (adapted from Jackson et al., 2010).

Name	#Subunits	Function
eIF2	3 (αβγ)	Forms an eIF2–GTP–Met-tRNA_i_ ternary complex that binds to the 40S subunit, thus mediating ribosomal recruitment of Met-tRNA_i_
eIF3	13	Binds 40S subunits, eIF1, eIF4G, and eIF5; stimulates binding of eIF2–GTP–Met-tRNA_i_ ternary complex (TC) to 40S subunits; promotes attachment of 43S complexes to mRNA and subsequent scanning
eIF1	1	Ensures the fidelity of initiation codon selection; promotes scanning; stimulates binding of TC to 40S subunit; prevents premature eIF5-induced hydrolysis of eIF2-GTP and *P*_i_ release
eIF1A	1	Stimulates binding TC to 40S subunit and cooperates with eIF1 in promoting scanning and start codon selection
eIF4E	1	Binds to the 5′ m^7^GpppG cap structure of mRNA
eIF4A	1	DEAD-box ATPase and ATP-dependent RNA helicase
eIF4G	1	Binds eIF4E, eIF4A, eIF3, PABP, and mRNA. Enhances elF4A helicase activity
eIF4F	3	Cap-binding complex, comprising eIF4E, eIF4A, and eIF4G; unwinds the 5′ proximal region of mRNA and mediates the 43S attachment; assists ribosomal complexes during scanning
eIF4B	1	An RNA-binding protein that enhances eIF4A helicase activity
eIF4H	1	Enhances the eIF4A helicase activity; is homologous to a fragment of eIF4B
eIF5	1	A GTPase-activating protein, specific for eIF2-GTP, induces GTP hydrolysis upon initiation codon recognition
eIF5B	1	A ribosome-dependent GTPase that mediates 40S/60S subunit joining
eIF2B	5	A guanosine nucleotide exchange factor that promotes GDP–GTP exchange on eIF2

The recruitment of the PIC to the 5′ cap is mediated by the eIF4 factors (4E, 4G, and 4A), which form a trimolecular complex referred to as eIF4F. The smallest of these, eIF4E, binds directly to the cap and is conserved across the eukaryotic kingdom (Marcotrigiano et al., 1997a,b). It is a target for phosphorylation by MNK kinases (Fukunaga and Hunter, 1997; Wang et al., 1998), an event that correlates with increased translation rates (Scheper and Proud, 2002; Scheper et al., 2002), and is a limiting initiation factor in many cell types. Three different eIF4E-family members have been characterized in mammals, eIF4E1, eIF4E2 (4EHP, 4E-LP), and eIF4E3. They differ in their structural signatures and functional characteristics (Joshi et al., 2004). eIF4E1 (referred to hereafter as eIF4E) is the major form that regulates global translation rates. It is a proto-oncogene, in that its over-expression induces cellular transformation (Lazaris-Karatzas et al., 1990). This is also frequently observed in a range of human tumors (Anthony et al., 1996; Yi et al., 2013). It appears that over-expression promotes the translation of a number of key malignancy-related proteins such as matrix metalloproteinase 9, vascular endothelial growth factor and cyclin D1. These are characterized by the presence of highly structured 5′ TLs, an observation in-line with the model that structured RNAs impede eIF4E access to the cap (Koromilas et al., 1992). The eIF4E recruits the PIC via the scaffolding protein eIF4G and is blocked by a series of eIF4E binding proteins (4EBPs) whose activity is modulated by mammalian/mechanistic target of rapamycin complex 1 (mTORC1)-mediated phosphorylation. This couples global translation rates to the PI3K/mTOR pathway (Gingras et al., 2001). The eIF4E/4EBP interplay is at the heart of translational homeostasis (Yanagiya et al., 2012). eIF4G functions as a modular adaptor protein between eIF4E, eIF3, and the eIF4A RNA helicase. Its N-terminal extremity also contains an interaction domain for the poly(A) binding protein (PABP), a cytoplasmic protein that binds the poly(A) tail. This interaction with eIF4G provides a physical link between the 5′ and 3′ extremities of mature mRNA, an event that serves to enhance translational initiation rates (Gallie, 1998).

Once recruited to the cap, the PIC moves forward (5′–3′) along the mRNA, scanning for the first AUG. Generally, this movement is facilitated by the RNA helicase eIF4A in an ATP dependent manner assisted by the single stranded RNA binding proteins eIF4B/eIF4H. They serve both to stimulate helicase activity and to limit RNA re-association (Rogers et al., 2001). Studies suggest that eIF1/1A also play active roles during scanning, particularly on mRNAs with unstructured 5′ TLs (Pestova and Kolupaeva, 2002). This can occur without eIF4A induced ATP hydrolysis. It seems that eIF1 promotes scanning when non-AUG codons are positioned in the P site probably by stabilizing an open conformation within the mRNA binding cleft of the 40S. In addition, it impedes initiation on non-AUG codons by blocking the hydrolysis of the eIF2 bound GTP or blocking the release of Pi from partially hydrolysed eIF2-GDP-Pi. These control functions are lost when the ribosome encounters an AUG start codon upon which eIF1 dissociates from its position close to the P site (Algire et al., 2005; Maag et al., 2005).

The nucleotides immediately surrounding an AUG codon influence the efficiency of its recognition. The sequence 5′-ACCAUGG-3′ is referred to as the optimal Kozak (1986) context since it is associated with efficient initiation rates. If sub-optimal, scanning ribosomes will sometimes ignore the first AUG codon and continue to the next. This phenomenon, known as leaky scanning, can produce N-terminal truncated proteins or proteins from overlapping reading frames. It appears that context is read by eIF1 (Pisarev et al., 2006). Once the AUG is recognized by the Met-tRNAi, the small ribosomal subunit pauses and a number of initiation factors are released. The eIF5B-GTP factor is recruited and the 60S subunit joins. GTP hydrolysis in eIF5B enables the release of eIF1A from the A site and eIF5B-GDP itself (Choi et al., 2000; Fringer et al., 2007). The 80S ribosome then enters the elongation phase of protein synthesis.

## The Mammalian 5′ Tl

### Elements That Modulate Translation Initiation

Loading of the PIC onto an mRNA and the subsequent identification of the initiation start site are the key events that dictate the translational readout both quantitatively and qualitatively. Central in both these events is the 5′ TL which carries features that will modulate both PIC recruitment and start site selection.

#### uAUGs/uORFs

Approximately 50% of human 5′ TLs contain one or multiple uORFs (Ingolia et al., 2009; Andreev et al., 2015). Despite their apparent abundance, uAUGs are less frequent than would be predicted by chance, yet AUG is the most conserved triplet within TLs. This suggests a strong evolutionary selection (Churbanov et al., 2005; Iacono et al., 2005). Moreover, ribosomal profiling studies performed on harringtonine treated cells (this drug blocks elongation and effectively freezes the 80S ribosome at its start site) suggest that this may be even more extensive due to the significant utilization of non-AUG initiation codons (e.g., CUG, GUG, UUG, and ACG) in mammals. These generate new uORFs when located within the 5′ TL, but can also give rise to multiple N-terminal protein isoforms when in-frame with the main ORF, or novel proteins when driving expression from an internal overlapping ORF (ioORF: see below; Ingolia et al., 2011; Ivanov et al., 2011). Both uAUGs and uORFs are generally viewed as translational repressors since they limit ribosome access to the downstream start codon for the main gene product. The amplitude of this repression is dictated by the context of the uAUG (Morris and Geballe, 2000). In addition to leaky scanning, the uAUG can also be bypassed by ribosomal shunting (Yueh and Schneider, 1996; Hohn et al., 1998; Latorre et al., 1998). However, small uORFs can also couple the readout to TC levels in the cell (Andreev et al., 2015) and, via the mechanism of delayed reinitiation, can permit access to start codons downstream of the AUG of the principle ORF (Rahim et al., 2012).

The efficiency of reinitiation responds to parameters such as uORF length and the distance between the stop codon and the next AUG (Peabody and Berg, 1986; Kozak, 1987; Rajkowitsch et al., 2004; Rahim et al., 2012). Reacquisition of the Met-tRNA by the 40S post-termination is dependent on eIF2-GTP levels. When it is low, the slow reacquisition can cause bypass of a proximal downstream AUG. The best characterized example of this phenomenon is the yeast GCN4. Translation of this gene occurs via reinitiation from a series of uORFs. During amino acid starvation, the eIF2α kinase GCN2 is activated and its phosphorylation of the α subunit of eIF2 impairs GTP exchange (phospho-eIF2-GDP is a competitive inhibitor of eIF2B). As a consequence, after translation of the first uORF the 40S bypasses a series of proximal downstream uORFs and initiates on the AUG^GCN4^ (Dever et al., 1992; Marton et al., 1997). HRI, PKR, PERK are other eIF2α kinases that are able to modulate reinitiation under various conditions of cellular stress or apoptosis. Collectively they are referred to as the “stress activated protein kinases” (Proud, 2005). A vaguely understood feature of the GCN4 5′ TL was the very divergent capacities of the four uORFs to permit efficient reinitiation. Sequences flanking the highly efficient uORF1 were reported to facilitate continued scanning post-termination (Grant et al., 1995). Recent studies suggest that eIF3, positioned near the mRNA exit channel on the ribosome, is retained during the scanning of uORF1. An interaction between eIF3a and an upstream enhancer element on the mRNA serves to stabilize the mRNA-40S association post-termination. This facilitates the resumption of scanning and downstream reinitiation (Szamecz et al., 2008; Munzarová et al., 2011; Beznosková et al., 2015). In a similar vein, the eIF3h subunit has been implicated in scanning, start site selection and reinitiation events in *Arabidopsis* (Choi et al., 2004; Roy et al., 2010). This opens the possibility that changes in the sequences flanking a uORF can impact on the read-out. Thus reinitiation in combination with leaky scanning offers the possibility to significantly increase the complexity of the mammalian proteome and both are clearly “tuned-in” to the physiological status of the cell. For example, the transcription factor CCAAT/enhancer binding protein β (C/EBPβ) mRNA expresses both long (LAP, liver activating protein) and N-terminally truncated short (LIP, liver inhibitory protein) isoforms via reinitiation events downstream of an uORF of 11 codons that terminates 4 nts before the LAP^AUG^ start codon. The N-terminal extension present on LAP contains trans-activating domains that induce differentiation and inhibit proliferation. Changes in the LAP/LIP ratio have been associated with human pathologies including cancer (Wethmar et al., 2010a,b). In a similar vein, our own work has demonstrated that reinitiation coupled to leaky scanning is employed to regulate the expression of the *ELK-1* gene and these events are fine-tuned by the alternative splicing of an exon within the 5′ TL that is positioned just upstream of a small uORF (Araud et al., 2007; Rahim et al., 2012; Legrand et al., 2014).

Apart from modulating the translational readout via delayed reinitiation, the major effect of cellular stress is to trigger a rapid down-regulation of global protein synthesis. Overall, this process is referred to as the integrated stress response (ISR) and the proteins that continue to be expressed during this phase will ultimately determine cell fate, i.e., recovery or apoptosis. The translational brake reflects increased eIF2α phosphorylation, an inhibition in TC regeneration and a subsequent reduction in the pool of 43S ribosomes. Until recently, it was widely accepted that the inhibitory and reinitiation phenotypes associated with the ISR were mechanistically coupled in that both arose due to a simple reduction in TC levels. However, recent studies from our lab suggest that it may not be so simple (Legrand et al., 2015). Using the phosphomimetic eIF2αS/D we could genetically differentiate these two processes in N2a cells (a neuroblastoma cell line). Whereas transient expression of eIF2αS/D could be shown to impact negatively on global protein expression it failed to modulate reinitiation (monitored using a number of specific reporters developed in the lab) and failed to trigger the ISR. To explain these observations we proposed that recruitment of the TC by the free 40S was different from recruitment by the 40S paused on the mRNA after translating a uORF. This may reside with the continued presence of initiation factors on the RNA-associated 40S in the ‘reinitiation mode’ (see above); factors that the free 40S subunit has lost and must recruit from the cytoplasmic pool. However, in HEK293T cells eIF2αS/D faithfully mimicked eIF2α phosphorylation, down-regulating global protein expression, modifying the reinitiation phenotype and triggering ISR. This suggests that the reinitiation machinery includes features that are cell-type specific.

#### RNA Structure

Highly structured 5′ TLs are frequently observed in the transcripts of genes whose protein products impact on the regulation of cellular proliferations and differentiation. As such it is a characteristic signature associated with tight translational control. Structure impacts on the protein readout at multiple levels. When positioned close to the 5′ it can render the cap less accessible, consequently these mRNAs compete poorly for the limiting amounts of eIF4E (Pickering and Willis, 2005). Moreover, bioinformatic studies suggest that structure near the 5′ cap may also play a role in miRNA mediated regulation possibly by blocking 43S scanning by interfering with the function of the initiation factor eIF4A2, a dead-box helicase paralog of eIF4A1 (referred to as eIF4A in the earlier section; Meijer et al., 2013; Gu et al., 2014). However, in a more recent alternative model, miRNA translational repression was proposed to act at the level of 43S loading onto the mRNA rather than subsequent scanning (Kuzuoğlu-Öztürk et al., 2016). This would be consistent with reports that the knockout of eIF4A2 in human cells did not suppress silencing (Galicia-Vazquez et al., 2015). Structure can also act post-43S recruitment, as a thermodynamic barrier that impedes ribosome movement during scanning. Its importance as a regulatory element is highlighted by the role of RNA helicases in human pathologies in which translational control is perturbed (Robert and Pelletier, 2013). Apart from eIF4A, other RNA helicases implicated in translational control include DHX29, DHX9 (also referred to as RNA helicase A or RHA) and DDX3. DHX29 is a DEAH-box protein that was initially reported to increase translation levels during cancer cell proliferation (Parsyan et al., 2009), promoting initiation on mRNAs with moderate to strong 5′ TL secondary structure (ΔG < -19 kcal/mol). It associates with the 40S subunit but is not found in polysomes, a feature characteristic of an initiation factor. Current models suggest, that rather than unwinding mRNA structure (it has a very poor processive helicase activity), DHX29 alters 40S conformation rendering it more processive (Pisareva et al., 2008). This is achieved by DHX29 cycling between NTP and NDP bound states and, in-so-doing, opening and closing the mRNA entrance site on the 40S. DHX29 in co-operation with eIF1A also plays a role in the regulation of leaky scanning and AUG codon selection (Pisareva and Pisarev, 2016). RHA has been implicated in transcription, miRNA biogenesis, splicing and nuclear export and may serve to couple nuclear and cytoplasmic events (Lee and Pelletier, 2016). It acts specifically on a subset of mRNAs by binding to, and unwinding, a structure in their 5′ TLs called the 5′ post-transcriptional control element (PCE; Hartman et al., 2006). This element is orientation dependent and has been observed in a number of retroviruses and the cellular c-JUND mRNA (Short and Pfarr, 2002). The PCE model posits that the RHA protein binds directly to the structural motif in the 5′ TL. However, other reports suggest that this may not always be the case. For example, Lin28 is an RNA binding protein important during development, pluripotency, and oncogenesis. It also stimulates the translational expression of a subset of mRNAs. It appears that this is achieved by Lin28 recruiting RHA to the mRNA (Jin et al., 2011). Thus RHA may impact on initiation at two levels, by direct binding to RNA structural motifs and, by recruitment via a second RNA binding trans-acting factor. In each scenario it would serve to promote the expression of specific mRNA sub-populations. Likewise, DDX3 has also been reported to selectively enhance the translation of specific mRNA populations characterized by the presence of RNA structural elements close to the cap. Such elements normally impede eIF4F binding and subsequent 43S loading. It appears that DDX3 associates with eIF4G within eIF4F and in combination with its intrinsic RNA binding activity serves to unfold the 5′ proximal RNA structure thereby facilitating entry of the 43S (Soto-Rifo et al., 2012).

Extensive structure within the 5′ TL is also a possible signature for IRES activity. IRESes were first characterized in picornaviruses. However, unlike their viral counterparts cellular IRESes tend to have low activity in normal growing cells probably because they compete poorly with the 5′cap for the PIC. Cellular IRES trans-acting factors (ITAFs) modulate activity in large part by serving as chaperones that ensure the correct RNA folding (Hunt et al., 1999; Pichon et al., 2012). Many cellular IRESes become activated during stress probably because these conditions down-regulate cap-dependent translation thereby making available a larger pool of free ribosomes. The proteins expressed via an IRES element function to protect the cell from the stress, or induce apoptosis, suggesting that IRES activity may play a key role in the cell fate decision (Komar and Hatzoglou, 2011). The reported cellular IRESes have very diverse RNA structures and very variable thermodynamic stabilities. Likewise, not all highly structured 5′ TLs have IRES activity and some can actually recruit the PIC via either the cap or internally depending on the physiological status of the cell (Pinkstaff et al., 2001). In the case of the *XIAP* gene, alternative splicing within the 5′ TL generates two variants one of which recruits via the cap whilst the second has IRES activity. This splicing event is coupled to cellular stress and ensures continued XIAP expression under these conditions (Riley et al., 2010). [NOTE: in this review we have focused mainly on cap-dependent translation].

#### TOP mRNAs

These transcripts harbor a terminal oligopyrimidine (TOP) tract at their 5′ end consisting of a cytosine at the penultimate nucleotide position followed by a stretch of 4–14 pyrimidines (Avni et al., 1997). TOP mRNAs generally encode components of the translational machinery including ribosomal proteins and elongation factors (Meyuhas and Hornstein, 2000). Expression of these proteins is highly sensitive to stress and growth conditions, a coupling that is mediated via the mTORC1 and specifically its downstream effector 4EBP (Thoreen et al., 2012). The mechanistic details behind the specificity of the 4EBP-TOP mRNA regulation remain vague and has even been contested in other studies (Gandin et al., 2016).

#### SHORT 5′ TLs

AUG codons close to the 5′ end (<20 nts) are generally considered to be silent or very leaky (Kozak, 1991; Pestova and Kolupaeva, 2002). However, one group of cellular mRNAs with short 5′ TLs possess a TISU motif (Translation Initiator of Short 5′ UTR/TL). This sequence element impacts both on transcription and translation, being particularly enriched in mRNAs transcribed from TATA-less promoters. Because of their very short 5′ TLs (<12 nts), the AUG start codon is apparently accessed in a 5′ cap dependent manner without apparent scanning. As a consequence, initiation events are largely independent of the initiation factors eIF4A and eIF1 (Elfakess et al., 2011; Dikstein, 2012). The histone H4-12 mRNA also carries a short, 9 nts, 5′ TL but without a TISU motif. In this case the PIC is initially recruited to a structural element within the ORF before being transferred to a second structural element at the 5′ end which facilitates engagement with the cap (Martin et al., 2011).

**FIGURE 2:**
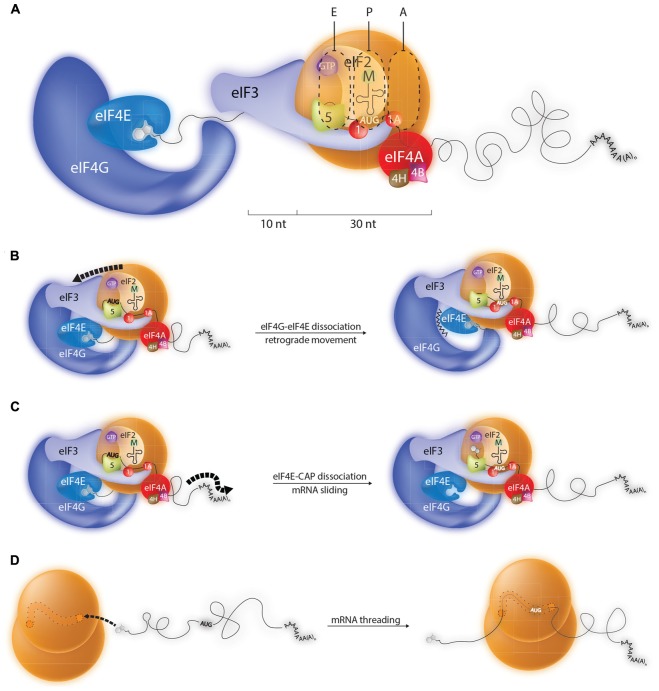
**Possible mechanisms by which the ribosome can recognize initiation codons close to the 5′ cap. (A)** During conventional scanning the 43S ribosome will move 5′–3′ on the mRNA until it positions an initiation codon in the P site. Probing studies indicate that the 43S paused over an AUG start codon makes contacts with around 40 nts of mRNA, 10 upstream nts of which are in close contact with eIF3. Such a configuration explains why AUG codons within the first 20 nts of most mammalian transcripts are poorly recognized by the ribosome. However, a number of models can be evoked to explain the initiation events observed on mRNA carrying TISU elements (translation initiation on short 5′ UTRs) which have 5′ TLs shorter than 10 nts. **(B)** Model 1: The eIF4E/4G contact is broken permitting retrograde movement (3′–5′) of the PIC. **(C)** Model 2: The eIF4E-5′ cap interaction is disrupted allowing the mRNA to slide over the surface of the 43S ribosome until the AUG enters the P site. **(D)** Model 3: Transcripts carrying 5′ TISU elements are selectively recruited to empty 80S ribosomes and then treaded through the mRNA channel until the AUG enters the P site.

Little is known about the molecular architecture of the 43S loaded onto the 5′ cap. Presumably, before scanning this complex is held in place by 5′ cap-eIF4E-eIF4G-eIF3-40S contacts (see **Figure 1**). Furthermore, we know absolutely nothing about how the mRNA sits on this 43S ribosome and what actually occurs to release the 43S from the 5′ end to permit scanning, if indeed it occurs. This has made it difficult to interpret the initiation events that one observes on short 5′ TLs. However, thanks to high resolution cross-linking studies we know considerably more about the path of the mRNA on the 48S positioned over a start codon that is 5′ distal (Pisarev et al., 2008). RNase protection studies (foot-prints) have shown that the mammalian 80S ribosome protects ∼30 nt of mRNA, but that the 48S subunit binds an additional 10–20 nt of RNA on the 5′ side (**Figure 2A**) (Kozak, 1977; Lazarowitz and Robertson, 1977). The cross-linking studies revealed that these additional protected nucleotides made numerous contacts with eIFs, particularly eIF3. Extrapolating to the ribosome recruited onto the 5′ cap, this is also consistent with the observation that efficient initiation requires a minimal TL length of 20 nts (distance between 5′ end and the 43S P site). This would, in-turn, suggest that the types of mRNA-eIF contacts observed in the 48S complex over an AUG are conserved in the 43S at the 5′ cap. So how does one position the AUG of a short 5′ TL in the P site? One possibility is that upon 43S loading the eIF4E-4G contact breaks, an event required for scanning. Biochemical data do suggest that the 4E-4G interaction is weakened when eIF4E binds the 5′ cap (Merrick, 2015). This would then permit retrograde movement of the PIC and positioning of the AUG in the P site (**Figure 2B**). Indeed, 3→5′ scanning has recently been evoked (Zinoviev et al., 2015). Furthermore, eviction of a part of the cap binding complex, possibly eIF4G in association with a fraction of eIF1, has been proposed to occur during initiation on TISU elements (Sinvani et al., 2015). However, how this process is driven remains unresolved. Alternatively, the eIF4E- 5′ cap interaction could be broken (HOW?) allowing the mRNA to slide over the surface of the small ribosome until the AUG enters the P site (**Figure 2C**). In another model direct loading is via the mRNA 5′ end on empty 80S particles free of eIFs (**Figure 2D**). The mRNA would then be treaded through the ribosome until the AUG enters the P site. Translation initiation with bacterial 70S monosomes on leaderless mRNAs has been known for some time (Moll et al., 2004). It is also known that eukaryotic cells contain a large pool of “empty” 80S monosomes that are considered to be biologically inactive (Krokowski et al., 2011). Nonetheless, it has been reported that purified liver monosomes can bind mRNA, aminoacylated tRNAs and drive peptide bond formation (Budkevich et al., 2008).

At another level, the translational readout can also be modulated by trans-acting cellular proteins binding to specific RNA features within the 5′ TL. This includes the IRPs (iron-regulatory proteins), which bind to stem–loop structures called the IRE (Medenbach et al., 2011; Sanchez et al., 2011), but it also appears that RNA binding proteins can modulate initiation events downstream of small uORFs (Medenbach et al., 2011). We, and others, have proposed that proteins interacting with the 5′ TL may facilitate 40S recruitment to the 5′ cap, possible by interacting with components of the PIC (Genolet et al., 2008, 2011; Gilbert, 2010).

### The Implications of 5′ Heterogeneity for the Proteome

Alterations in the nature of the 5′ TL arise due to the use of alternative promoters, TSS heterogeneity and alternative splicing. These serve to couple events in the nucleus to the proteomic readout in the cytoplasm. In particular, extensive heterogeneity exists due to multiple TSSs (Stamatoyannopoulos, 2010; Pal et al., 2011). This is even observed in yeast in which over 26 major transcript variants were detected per protein coding gene (Pelechano et al., 2013). In mice, the number of detected transcripts is at least one order of magnitude greater than the ∼22,000 genes of the genome (Carninci et al., 2005). There are two well established modes of transcription initiation, referred to as focused and dispersed (Danino et al., 2015). Focused promoters have relatively few TSSs falling within a narrow region defined as between -40 and +40 nts relative to the major start site, and generally have a TATA box. On the other hand, dispersed promoters have multiple weak start sites spanning over 100 nts. Over 70% of vertebrate promoters are dispersed and they generally possess CpG islands and Sp1/NF-Y transcription factor binding sites. Hence extensive 5′ TL heterogeneity is the norm for the mammalian transcriptome. Furthermore, complete switches in the dominant TSS have been reported to occur in over 300 genes during differentiation with more subtle shifts being detected in over 1,300 others (Trapnell et al., 2010), suggesting that the TSS fingerprint (the relative abundance of the TSS variants of each gene promoter) within the transcriptome is a genetic marker for cellular type (phenotype). In a recent study, 5′ rapid amplification of cDNA ends (5′ RACE) was coupled to next generation sequencing (NGS) to probe TSS variability at the glucocorticoid receptor (GR) promoter. They observed 358 TSSs that altered the nature of the 5′ TL. Furthermore, promoter activation with dexamethasone and γ-interferon added an additional 185 new TSSs distributed throughout the promoter region. *In vitro* studies indicated that this TSS variability impacted on translation efficiency and abundance of the different GR N-terminal protein isoform levels (Leenen et al., 2016). However, the impact of global changes on TSS selection on the mammalian proteome remains unclear despite evidence from yeast that it correlates with a major shift in translational activity (Rojas-Duran and Gilbert, 2012). The species conservation of elements that are thought to play a role in translational control, suggest that these variations are functionally relevant (Churbanov et al., 2005). These include length, RNA structure, uAUGs, uORFs, and IRES elements.

It is evident that the aberrant use of alternative promoters that alter the translational read-out, can impact significantly on normal cellular function (Davuluri et al., 2008; Singer et al., 2008). Complete gene promoter switches have been linked to a number of human pathologies (Calvo et al., 2009; Somers et al., 2013). Frequently they alter protein expression due to the presence of uORF(s)/uAUG(s) in one of the transcript variants. For example, the *MDM2* gene, whose major protein product serves to regulate p53 levels in the cell, uses two alternative promoters that express transcripts with a long and a short TL. Initiation at the AUG^MDM2^ in the long TL is repressed due to the presence of two uORFs. These elements are absent in the short TL variant. In certain tumors, mdm2 protein over-expression arises due to the enhanced expression of the short form. Additionally, polysome analysis suggests that the short TL variant is more efficiently translated in tumoural cells (Brown et al., 1999), demonstrating that the impact of a promoter switch can then be amplified by the cell-specific seeding of a specific TL variant onto polysomes. Our own studies have indicated that the uORFs present within the long TL also promote reinitiation events that qualitatively change the protein read-out (Genolet et al., 2011). Furthermore, when examining promoter switches one must also consider changes in the TSS fingerprint. This is particularly pertinent for transcripts with AUG codons positioned close to the 5′ end since TSS heterogeneity could impact significantly on their utilization as a start codon. Another twist on this story is that promoter usage can also impact on splicing patterns and transcription TTSs (Oktaba et al., 2015; Slobodin and Agami, 2015). Changes in the nature of the 3′ UTR could then impact on miRNA mediated control.

#### Cap Analysis of Gene Expression (CAGE) Analysis

The +1 position on the mammalian mRNA is notoriously poorly annotated, an observation that reflects a 3′ bias in the early database. However, this is currently being rectified as a result of high-throughput cap analysis of gene expression (CAGE; Kodzius et al., 2006; Takahashi et al., 2012). CAGE libraries contain 27 nts tags corresponding to the very 5′ ends of capped RNAs. They are therefore ideal for characterizing TSS complexity. The FANTOM5 Promoterome database annotates 5′ cap tags generated by CAGE (The Fantom Consortium et al., 2014). As yet, this approach has not been exploited to analyze TSS complexity within the translatome (polysome associated mRNAs). Furthermore, to define the complete 5′ TL the short tag must overlap the RefSeq or Ensembl annotations. CAGE is also complemented by the Eukaryotic Promoter database (EPD^1^), a collection of promoter mapping experiments from multiple species generated by NGS (Dreos et al., 2015).

#### Ribosome Filter Hypothesis

Not all the information for decoding an mRNA resides within its sequence. Indeed, the translational readout from a specific transcript can show considerable cell-type specificity. This is explained, at least in-part, by the activity/availability of trans-acting factors such as RNA helicases, RNA binding proteins and eIFs. Nonetheless, it is evident that the ribosome machine itself can function as a molecular filter, selecting specific transcript sub-populations to seed the polysome (Mauro and Edelman, 2002, 2007). In part, this operates via RNA–RNA interactions between the target transcript and the ribosome. These interactions could be modulated by RNA binding proteins that mask a site, or chaperon RNA folding. This latter feature explains the function of ITAFs (Bushell et al., 2006; Anderson et al., 2007). The filter would respond to proliferative, developmental and environmental signals and its dysfunctioning could be a key element in a number of physiological disorders (ribosomal pathologies; Cazzola and Skoda, 2000; Narla and Ebert, 2010). Indeed, in a transcriptome/translatome study using a glioblastoma model, the authors concluded that the selective polysomal recruitment of specific mRNA populations could initiate and drive tumor formation (Rajasekhar et al., 2003). The filter would also be regulated by features within the core ribosomal machine. These could arise as a result of heterogeneity in cellular ribosomal protein levels, which may reflect transcriptional and/or post-transcriptional changes, in combination with covalent modifications of the ribosomal proteins and rRNA (Sussman, 1970; Mauro and Edelman, 2002, 2007). In this model, the cellular pool of ribosomes is not homogeneous but is rather a heterogeneous assembly of variable composition each with a specific preference for features within its mRNA target. Considering the importance of the mammalian 5′ TL for ribosome loading it seems reasonable to propose that the complexed 5′ heterogeneity observed within the transcriptome will in-turn be filtered, in a cell-specific manner, to seed a different and distinct 5′ TL fingerprint within the translatome. To test this we employed RNAseq to characterize both the transcriptome and translatome of the tumoural MCF7 tumoural and non-tumoural MCF10A cell lines. By focusing on genes exhibiting well annotated TSS heterogeneity, we noted distinct differential promoter usage patterns in the transcriptome and translatome, a result consistent with a cell-specific ribosome filtering of the transcriptome (Dieudonné et al., 2015).

### Curiosities of the Protein Readout

#### Peptides Encoded by uORFs

Genome/transcriptome and proteome analysis has identified thousands of as yet non-annotated short open reading frames (smORFs) with the potential to encode biologically active peptides (Saghatelian and Couso, 2015). One source of these is the uORFs within the 5′ TL. Some can encode functional proteins (Andrews and Rothnagel, 2014; Andreev et al., 2015). These short peptide sequences can act either *in-cis* to modulate downstream initiation events, or have distinct biological function(s). An interesting group of these *cis*-acting peptides are responsive to environmental signals and have been coined “peptoswitches” (Jorgensen and Dorantes-Acosta, 2012). The signals generally take the form of small molecular metabolites such as sugars or amino acids that interact with the nascent small peptide chain causing a ribosome pause.

#### Internal Overlapping ORFs (ioORFs)

Leaky scanning and delayed reinitiation also permit access to internal AUG codons. When in-frame with the principle ORF they give rise to N-terminally truncated proteins (Ivanov et al., 2011; Van Damme et al., 2014). When positioned out-of-frame (ioORF), they represent a second source of smORFs. The expression of biologically active proteins from ioORFs has actually been known for some time. It was described in mammalian viral systems as far back as the early 1980’s (Giorgi et al., 1983; Curran et al., 1986). Nevertheless, its implications for the human proteome are only now beginning to be appreciated (Mouilleron et al., 2016). For example, within the ataxin-1 (*ATXN1*) transcript a small ioORF starting 30 nts downstream of the AUG^ATXN1^and in the -1 reading frame is expressed by leaky scanning (Bergeron et al., 2013). The protein, referred to as Alt-ATXN1, was observed to co-localize and interact with Ataxin-1 within nuclear inclusions. In a similar vein, the prion protein gene *PRNP* also expresses a novel polypeptide from an ioORF, referred to as AltPrP (Vanderperre et al., 2011). It localized at the mitochondria and was up-regulated by ER stress and proteasomal inhibition. Using polyclonal antiserum it was detected in human brain homogenates, primary neurons, and peripheral blood mononuclear cells. Despite sizes smaller than 100 aas, the products of smORFs may have important biological functions. In mice, the Mln smORF expresses a 46 aa peptide that plays a role in muscle contraction whereas in humans the Humanin smORF (24 aas) is implicated in apoptosis and the MRI-2 smORF (69 aas) in DNA repair (Saghatelian and Couso, 2015). With regards to clinical medicine, a number of human cancer specific antigens are also expressed from iORFs (Slager et al., 2003; Oh et al., 2004). Their expression reflects the change in the translational landscape that occurs with cellular transformation and they represent novel targets for immune based therapies.

#### What Ribosome Profiling Tells us

Detecting the products of smORFs, which may be numerous and small (<100 aas), is technically not straightforward (Couso, 2015). However, identification has been facilitated by ribosome profiling (Ingolia et al., 2009). This technique couples ribosome footprinting to high-throughput RNAseq providing quantitative information about ribosome density across a gene transcript. It has been used to identify alternative START/STOP sites, initiation from non-AUG codons, translational pausing/frame-shifting as well as expression from uORFs or over-lapping iORFs (ioORFs; Mumtaz and Couso, 2015). However, one limitation of the technique is that it can provide little information about the nature of non-translated *cis*-acting regulatory sequences that may reside within the 5′ TL and 3′ UTR. Consequently, changes in the translational readout coupled to promoter switches, TSS heterogeneity or alternative splicing may not be detected (see below).

## Recapitulation

Despite major advances in our understanding of how the *cis*-regulatory elements residing within the mammalian 5′ TL modulate the protein readout we are only beginning to understand the impact of transcriptional heterogeneity on this process. In this review we have focused mainly on the promoter. However, another twist on this story is that promoter usage can impact on splicing patterns and transcription TTSs, events that can in-turn also modulate the protein readout (Oktaba et al., 2015; Slobodin and Agami, 2015). For example, changes in the nature of the 3′ UTR can impact on miRNA mediated control. Thus we find ourselves scratching at the surface of a new layer of complexity in the regulation of gene expression and the cellular phenotype.

## Author Contributions

The text was in large part written by JC after numerous discussions with BW. All graphic art was prepared by BW.

## Conflict of Interest Statement

The authors declare that the research was conducted in the absence of any commercial or financial relationships that could be construed as a potential conflict of interest.

## Footnotes

1 http://epd.vital-it.ch
